# Serum albumin and mortality in patients with HIV and end-stage renal failure on peritoneal dialysis

**DOI:** 10.1371/journal.pone.0218156

**Published:** 2019-06-10

**Authors:** Kwazi Celani Zwakele Ndlovu, Perpetual Chikobvu, Thabiso Mofokeng, Verena Gounden, Alain Assounga

**Affiliations:** 1 Division of Nephrology, University of the Free State, Bloemfontein, South Africa; 2 Department of Internal Medicine, University of the Free State, Bloemfontein, South Africa; 3 Department of Health of the Free State, Bloemfontein, South Africa; 4 Department of Community Health, University of the Free State, Bloemfontein, South Africa; 5 Department of Chemical Pathology, Inkosi Albert Luthuli Hospital, Durban, South Africa; 6 Department of Nephrology, University of KwaZulu-Natal, Durban, South Africa; Universidade de Sao Paulo Faculdade de Medicina, BRAZIL

## Abstract

**Background:**

Peritoneal dialysis (PD) is an easily implementable dialysis modality in end-stage renal disease (ESRD). PD may improve access to renal replacement therapy in low- and middle-income countries; however, these countries have a higher prevalence of protein-energy wasting in patients and poorer socioeconomic conditions. We evaluated the effects of HIV infection on serum albumin levels in ESRD patients starting continuous ambulatory PD (CAPD) and mortality outcomes.

**Methods:**

We conducted a single-center prospective cohort study of consecutive incident CAPD patients recruited from two hospitals in Durban, South Africa, from September 2012 to February 2015. Seventy HIV-negative and 70 HIV-positive ESRD patients were followed monthly for serum albumin levels and mortality events during the first 18 months of CAPD therapy.

**Results:**

The HIV-positive cohort recorded 28 deaths (40%) among patients with a functional CAPD catheter at 18 months and 13 deaths (18.6%) in the HIV-negative cohort (*p* = 0.005). The mean serum albumin levels were lower in the HIV-positive cohort than in the HIV-negative cohort during the 18-month follow-up. The mean difference in serum albumin levels between the two cohorts was 4.24 g/L (95% confidence interval [CI] 2.02–6.46, *p*<0.001) at baseline and 3.99 g/L (95% CI 1.19–6.79, *p* = 0.006) at 18 months. HIV-positive status (adjusted regression coefficient -2.84, CI -5.00–-0.67, *p* = 0.011), diabetes (adjusted coefficient -2.85; CI, -5.58–-0.12; *p* = 0.041), and serum C-reactive protein and blood hemoglobin levels were independent predictors of serum albumin levels on multivariable linear regression. Baseline serum albumin <25 g/L (subdistribution-hazard ratio [SHR] 13.06, 95% CI 3.09–55.14, *p*<0.001) and CD4^+^ cell count <200 cells/μL (SHR 3.2, CI 1.38–7.45, *p* = 0.007) were independent predictors of mortality in our competing risk model.

**Conclusions:**

HIV infection can adversely affect serum albumin levels in ESRD patients managed with CAPD, while low baseline serum albumin levels and impaired immunity reliably predict mortality.

## Introduction

Peritoneal dialysis (PD) is an easily implementable renal replacement therapy (RRT) option for patients with end-stage renal disease (ESRD) owing to fewer resource requirements compared to hemodialysis and has been suggested to be a cost-effective approach when favorable country policies underpin its application [1–5]. In low- and middle-income countries that typically have reduced access to RRT due to poor health care funding and infrastructure, PD can potentially allow for easier upscaling of RRT access when local manufacturing and policy conditions favor lower costs of PD fluids [1, 6, 7].

Protein-energy wasting (PEW) is an important complication in ESRD, and a number of factors have been recognized to contribute to its development, such as uremia, persistent inflammation, metabolic acidosis, endocrine abnormalities, dialysis-associated losses, and comorbid conditions such as diabetes mellitus and human immunodeficiency virus (HIV) [8–12]. PD can exacerbate PEW due to losses in protein, amino acids, and other nutrients into the dialysate as well as inadequate nutritional intake, due to abdominal distension caused by the continuous inflow of dialysis fluid, and early satiety caused by the constant absorption of glucose [13–15]. Comorbid infection with HIV can further complicate the incidence of PEW in ESRD patients managed with PD especially since the majority of the HIV-positive population resides in sub-Saharan Africa, an area associated with high rates of poverty and undernutrition [16]. HIV can be complicated with significant malnutrition that may not improve sufficiently even on commencement and maintenance with antiretroviral treatment (ART) [17, 18].

Serum albumin is a readily available and convenient biomarker that has traditionally been used as an indicator of nutritional status and protein balance in ESRD patients on dialysis [14, 19]. However, many non-nutrition factors have also been recognized to affect serum albumin levels such as systemic inflammation, volume status, and comorbidities [20, 21]. Serum albumin has been shown to have a predictive value with regard to morbidity and mortality for both hemodialysis and PD [19, 22, 23]. Its ability to predict illness and death has been shown to be strongly influenced by inflammation [24, 25]. Persistent systemic inflammation may act by disturbing the balance established between losses associated with ESRD and dialysis modalities and compensatory increases in hepatic albumin synthesis [23, 26]. HIV infection is associated with ongoing immune activation and chronic inflammation that may not improve even on ART initiation [27, 28]. However, the effect of HIV on the complex interactions between malnutrition and inflammation associated with ESRD patients managed with PD and related patient outcomes are not well defined. This study aimed to evaluate the effects of HIV-seropositive status on serum albumin levels in ESRD patients started on continuous ambulatory peritoneal dialysis (CAPD) and the effects of HIV and baseline serum albumin levels on mortality.

## Materials and methods

### Patients and study design

This prospective cohort study was conducted on 140 patients, comprising 70 HIV-positive and 70 HIV-negative patients recruited from the King Edward VIII Hospital and Inkosi Albert Luthuli Central Hospital (IALCH), Durban, South Africa, as described previously [29, 30]. Consecutive incident patients with end-stage renal failure aged 18–60 years with newly inserted double-cuffed coiled Tenckhoff catheters were recruited between September 2012 and February 2015. Enrolment was stopped when each cohort totaled 70 participants. The University of KwaZulu-Natal Biomedical Research Ethics Committee (BE 187/11) approved the study protocol, and informed consent was obtained from all patients before enrolment. All clinical activities and investigations were conducted in accordance with the guidelines of the 2008 Declaration of Helsinki.

The status of HIV infection was determined by two 4th generation HIV enzyme-linked immunosorbent assays performed by the South African National Health Laboratory Service (NHLS) before enrolment. Screening for HIV was performed using an HIV Ag/Ab Combo (CHIV) assay (ADVIA Centaur XP, Siemens Healthcare Diagnostics, Tarrytown, NY, USA), and confirmation testing was performed using HIV Combi and HIV Combi PT assays (Cobas e601, Roche Diagnostics, Mannheim, Germany). All HIV-positive patients were on ART, and management thereof was left to the discretion of the local clinic guided by South African national protocols. Y-sets, twin-bag systems, and conventional peritoneal dialysis (PD) solutions (Dianeal 1.5%, 2.5%, or 4.25% dextrose, icodextrin, or 1.1% amino acid-based solutions; Baxter Healthcare, Deerfield, IL, US) were used in all CAPD patients, and generally, four exchanges per day were performed. A 1.1% amino acid-based peritoneal dialysis solution (Nutrineal) was available to patients with serum albumin levels <20 g/L replacing one dextrose exchange during the day, and a renal dietician was available for dietary counseling and planning to all patients.

### Enrolment and follow-up

Patients’ demographic, clinical, and biochemical data were documented on enrolment. Patients were followed monthly at a central renal clinic at the IALCH for 18 months or up to catheter removal and subsequent transfer to hemodialysis or death. At each monthly follow-up assessment, phlebotomy was performed for full blood counts, and serum concentrations of C-reactive protein (CRP), urea, creatinine, electrolytes, and albumin were measured at the NHLS. Biochemical tests were performed using the ADVIA 1800 Clinical Chemistry System (Siemens AG, Munich, Germany), and the method used for analysis of serum albumin was spectrophotometric assay using a BCG dye binding method. Cluster of differentiation 4 positive (CD4+) T cell counts were assessed at baseline and every 6 months on HIV-positive patients. The results were periodically retrieved from the IALCH’s electronic results database and recorded on predefined questionnaires.

### Mortality

The in-hospital mortality occurring at the IALCH was recorded with details of causes and dates of incidence. Details of home deaths (occurring outside of IALCH) were obtained via telephone interviews with each participant’s relatives.

### Statistical analysis

Continuous variables are expressed as mean ± standard deviation (SD) or median and interquartile range (IQR), and they were compared using Student’s t-test or Wilcoxon–Mann–Whitney test, as appropriate. Proportions and categorical variables were compared using the chi-square test or Fisher’s exact test, as appropriate. A two-factor analysis of variance for repeated measurements was used to compare monthly serum albumin means over time according to HIV status. Linear regression clustered at the individual level was used to assess the relationship between HIV and serum albumin levels. A clustered multivariable linear regression model that included HIV status, age, race, sex, smoking, alcohol use, body mass index (BMI), month of follow-up the albumin specimen was taken, Tenckhoff catheter insertion parameters (site and method, whether laparoscopic or percutaneous), type of primary residence (urban vs rural), highest education level, exposure to peritonitis during follow-up, blood hemoglobin levels, and the serum levels of bicarbonate, urea, creatinine, ferritin, and CRP during follow-up, was used to determine whether HIV could independently predict monthly serum albumin levels.

Patient survival estimates were computed using the Kaplan–Meier method, and the log-rank test was used to compare survival curves. The Fine and Gray proportional hazards model was used to estimate the association between HIV seropositive status and all-cause mortality with technique failure treated as a competing risk and censored for lost to follow-up. Covariates included in the competing risks model were baseline serum albumin levels, age, race, sex, smoking status, diabetes, BMI, Tenckhoff catheter insertion parameters (site and method, whether laparoscopic or percutaneous), type of primary residence (urban vs rural), highest education level, baseline CD4+ cell count, baseline blood hemoglobin level, and baseline serum levels of ferritin and CRP during follow-up. All analyses were performed using Stata Statistical Software, Release 15 (StataCorp, College Station, Texas, USA). The level of significance was set at *p*<0.05.

## Results

### Patient characteristics

The median patient age was 40 years (interquartile range [IQR], 29–49) for the HIV-negative cohort and 35 years (IQR, 30–42) for the HIV-positive cohort (*p* = 0.260). Half of HIV-positive patients were started on ART less than 6 months before the start of CAPD. However, 57.1% of HIV-positive patients had viral loads below the detection limit of the hospital laboratory assay (<150 copies/mL) at the time of enrollment. For patients with detectable viral loads the median baseline viral load was 4230 copies/mL (IQR, 903–91,143), whereas the median dropped below the detectable limit (IQR <150–2990) when patients with undetectable viral loads were included. Other details of the study population are outlined in Table 1 and have been previously described [29, 30].

**Table 1:** Baseline characteristics according to HIV serostatus.

Variable	HIV-negative (*n* = 70)	HIV-positive (n = 70)	*p*-value
Age, median (IQR)	40 (29–49)	35 (30–42)	0.260^d^
Body mass index, median (IQR)	24.0 (22.0–28.5)	23.0 (20.9–28.0)	0.210^d^
Sex			
Female, n (%)	30 (42.9)	37 (52.9)	0.236^b^
Ethnicity			
African, n (%)	59 (84.3)	70 (100.0)	0.001^c^
Indian, n (%)	9 (12.9)	0 (0.0)	
Mixed ethnicity, n (%)	2 (2.9)	0 (0.0)	
Hypertension, n (%)	63 (90.0)	52 (74.3)	0.015^b^
Diabetes, n (%)	4 (5.7)	7 (10.0)	0.532^c^
Hepatitis B, n (%)	7 (10.0)	8 (12.1)	0.737^b^
Primary residence			
Urban, n (%)	44 (62.8)	45 (64.3)	0.956^b^
Rural, n (%)	23 (32.9)	24 (34.3)	
Education level			
Primary school, n (%)	15 (21.4)	13 (18.6)	0.710^b^
High school, n (%)	32 (45.7)	31 (44.3)	
Post-grade 12, n (%)	20 (28.6)	25 (35.7)	
Smoking (currently)	4 (5.71)	7 (10.0)	0.532^c^
Tenckhoff catheter insertion method			
Laparoscopic, n (%)	66 (94.3)	35 (50.0)	< 0.001^c^
Percutaneous, n (%)	4 (5.71)	35 (50.0)	
Tenckhoff catheter insertion site			
IALCH, n (%)	66 (94.3)	50 (71.43)	0.001^c^
KEH, n (%)	4 (5.7)	20 (28.6)	
Serum albumin			
Mean (g/L ± SD)	35.3 ± 6.7	31.0 ± 6.6	<0.001^a^
Alb ≥ 35 g/L, n (%)	41 (58.6)	18 (25.7)	<0.001^c^
Alb 30–34.9 g/L, n (%)	16 (22.9)	17 (24.3)	
Alb 25–29.9 g/L, n (%)	10 (14.3)	25 (35.7)	
Alb <25 g/L, n (%)	3 (4.3)	10 (14.3)	
Hemoglobin (g/dL), median (IQR)	9.4 (8.2–10.9)	8.9 (7.8–9.8)	0.040^d^
eGFR (mL/min/1.73 m^2^), median (IQR)	6 (5–8)	6 (5–8)	0.993^d^
CRP (mg/L), median (IQR)	20 (6–36.5)	56.5 (21–108)	<0.001^d^
Ferritin (ug/L), median (IQR)	626 (335–1049)	593 (381–973)	0.960^d^
ESR (mm/h), median (IQR)	42 (29–61)	93 (59–129)	< 0.001^d^
CD4+ cell count (cells/μL), median (IQR)		356.5 (217–481)	
Viral load (copies/mL), median (IQR)		LDL (LDL–2990)	
ART history at enrollment			
<6 months, n (%)		36 (51.4)	
6–12 months, n (%)		9 (12.9)	
>1 year, n (%)		25 (35.7)	
ART drug regimens			
3TC/EFV/ABC, n (%)		59 (84.3)	
3TC/EFV/AZT, n (%)		2 (2.9)	
3TC/EFV/D4T, n (%)		3 (4.3)	
3TC/NVP/ABC, n (%)		3 (4.3)	
3TC/Aluvia/ABC, n (%)		1 (1.43)	
3TC/Aluvia/AZT, n (%)		1 (1.43)	

3TC, lamivudine; ABC, abacavir; Alb, albumin; ART, Anti-retroviral therapy; AZT, zidovudine; CD = cluster of differentiation; CRP, C-reactive protein; D4T, stavudine; EFV, efavirenz; eGFR, estimated glomerular filtration rate (MDRD equation); ESR, erythrocyte sedimentation rate; HIV, human immunodeficiency virus; IALCH, Inkosi Albert Luthuli Central Hospital; KEH, King Edward VIII Hospital; LDL, lower than detectable limit (<150 copies/mL); NVP, nevirapine

^a^Student’s t-test,

^b^Pearson’s χ^2^ test,

^c^Fisher’s exact test,

^d^Wilcoxon–Mann–Whitney test

### Serum albumin

**Fig 1:**
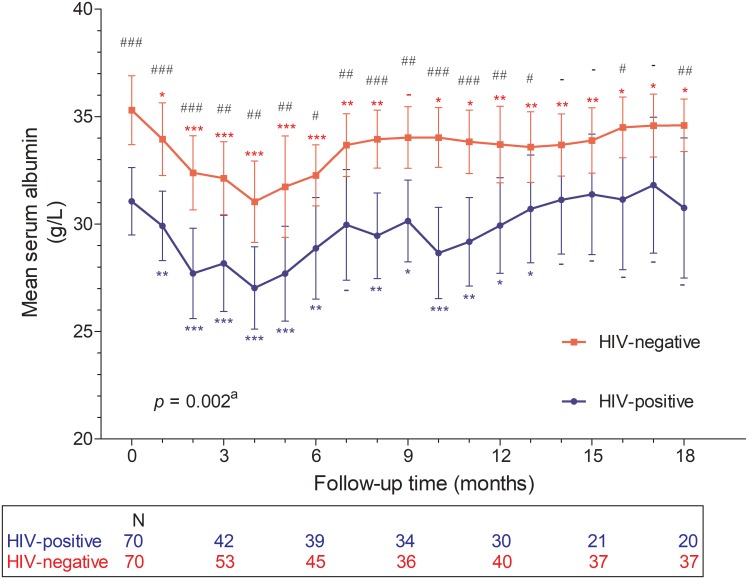
Monthly mean serum albumin with confidence intervals according to HIV-status. HIV, human immunodeficiency virus ^a^Two-factor analysis of variance for repeated measurements comparing monthly mean serum albumin over 18 months according to HIV status # independent t-test comparison of monthly mean serum albumin between the HIV-negative and HIV-positive cohorts *p*<0.05 (##, *p*<0.01; ###, *p*<0.001; -, *p*>0.05) * paired t-test comparison of means between baseline and follow-up monthly serum albumin *p*<0.05 (**, *p*<0.01; ***, *p*<0.001; -, *p*>0.05) N, number of study patients who presented and were sampled at specified clinic visit.

In this study, 41% (29/70) of the HIV-negative cohort and 74.3% (52/70) of the HIV-positive cohort had baseline serum albumin levels below 35 g/L (*p*<0.001) (Table 2). Mean serum albumin levels were significantly lower in the HIV-positive cohort than in the HIV-negative cohort during follow-up (S1 Table) (Fig 1). Paired monthly serum albumins means were significantly lower during the follow-up compared to the baseline for the first 13 months of follow-up in the HIV-positive cohort and throughout most of the 18 months in the HIV-negative cohort (Table 3) (S2 Table). Serum albumin levels decreased at similar rates in the HIV-negative cohort (linear regression coefficient (β), -1.048; 95% confidence interval [CI], -1.449–-0.647; *p*<0.001) and the HIV-positive cohort (β, -1.016; CI, -1.577–-0.456; *p* = 0.001) during the first 4 months of follow-up (difference in slopes (Δβ) 0.032; -0.649–0.712; *p* = 0.927). Likewise, both cohorts experienced comparable rates of increase between the 4th and 18th months of follow-up (β_HIV-negative_, 0.200; CI, 0.070–0.331; *p* = 0.003; β_HIV-positive_, 0.282; 95% CI, 0.077–0.487; *p* = 0.008; Δβ, 0.081; CI, -0.157–0.320; *p* = 0.499) (S1 Fig). HIV (crude regression coefficient -3.96; CI -5.79–-2.13; *p*<0.001) and diabetes (coefficient -5.06; CI, -7.72–-2.40; *p*<0.001) were significantly associated with monthly serum albumin levels on univariate linear regression analysis. Furthermore, both were independent negative predictors of monthly serum albumin levels on multivariable analysis (adjusted regression coefficient -2.84; CI, -5.00–-0.67; *p* = 0.011; and adjusted coefficient -2.85; CI, -5.58–-0.12; *p* = 0.041; respectively) (Table 4).

**Table 2:** Baseline characteristics according to serum albumin.

	Baseline serum albumin < 35 g/L	Baseline serum albumin ≥ 35 g/L	*p*-value
HIV-negative, n (%)	29 (41.4%)	41 (58.6%)	<0.001^a^
HIV-positive, n (%)	52 (74.3%)	18 (25.7%)	
Baseline CD4+ cell count (cells/μL)			
CD4+ <200, n (%)	13 (18.6%)	1 (1.4%)	0.036^b^
CD4+ 200–350, n (%)	17 (24.3%)	3 (4.3%)	
CD4+ ≥350, n (%)	22 (31.4%)	14 (20.0%)	
ART duration at enrolment			
ART <6 months, n (%)	32 (45.7%)	4 (5.7%)	0.006^b^
ART ≥6 months, n (%)	20 (28.6%)	14 (20.0%)	
Baseline plasma viral load			
VL <150 copies/mL, n (%)	28 (40.0%)	12 (17.1%)	0.343^a^
VL >150 copies/mL, n (%)	24 (34.3%)	6 (8.6%)	
Baseline CRP, median (IQR)	54 (20.5–106)	17.5 (5–37)	<0.001^c^
Baseline ESR, median (IQR)	77 (51–127)	38.5 (30–80)	<0.001^c^
Baseline HB, median (IQR)	8.3 (7.6–9.3)	10.0 (9.0–11.6)	<0.001^c^
Baseline ferritin, median (IQR)	627.5 (433.5–1146.5)	564 (196–1031)	0.078^c^
Baseline BMI, median (IQR)	23.7 (21.4–28.5)	23.6(20.8–26.5)	0.259^c^

ART, Anti-retroviral therapy; BMI, body mass index; CD = cluster of differentiation; CRP, C-reactive protein; ESR, erythrocyte sedimentation rate; HIV, human immunodeficiency virus; HB, blood hemoglobin; IQR, interquartile range; VL, viral load.

^a^Pearson’s χ^2^ test,

^b^Fisher’s exact test,

^c^Wilcoxon–Mann–Whitney test

**Table 3:** Comparison of mean serum albumin differences according to HIV status and between baseline and follow-up months.

	Independent t-test (HIV-negative vs. HIV-positive)		Paired t-test (baseline vs. monthly means)			
			HIV-negative		HIV-positive	
	MD (95% CI)	*p*-value	MD (95% CI)	*p*-value	MD (95% CI)	*p*-value
Baseline	4.24 (2.02–6.46)	<0.001	Reference		Reference	
2^nd^ month	4.68 (2.02–7.34)	<0.001	3.54 (1.88–5.20)	<0.001	4.47 (2.72–6.22)	<0.001
4^th^ month	4.01 (1.33–6.69)	0.004	3.89 (2.28–5.49)	<0.0001	5.35 (2.95–7.75)	<0.001
6^th^ month	3.39 (0.76–6.03)	0.002	4.27 (2.50–6.03)	<0.001	3.79 (1.25–6.34)	0.004
12^th^ month	3.77 (1.00–6.53)	0.008	3.05 (0.85–5.25)	0.008	3.37 (0.63–6.11)	0.018
18^th^ month	3.99 (1.19–6.79)	0.006	2.26 (0.20–4.33)	0.032	2.60 (-0.74–5.94)	0.120

CI, confidence interval HIV, human immunodeficiency virus; MD, mean difference

**Table 4:** Univariate and multivariable clustered linear regression analysis of monthly average serum albumin.

	Univariate linear regression		Multivariable linear regression^a^	
	Coef. (95% CI)	*p-*value	Coef. (95% CI)	*p-*value
HIV	-3.96 (-5.79–-2.13)	<0.001	-2.84 (-5.00–-0.67)	0.011
Diabetes	-5.06 (-7.72–-2.40)	<0.001	-2.85 (-5.58–-0.12)	0.041
Age	0.0004 (-0.10–0.10)	0.995	-0.02 (-0.14–0.09)	0.717
BMI	0.11 (-0.08–0.29)	0.264	0.09 (-0.12–0.30)	0.382
Serum CRP	-0.04 (-0.05–-0.03)	<0.001	-0.02 (-0.03–-0.01)	<0.001
HB	0.97 (0.66–1.28)	<0.001	0.68 (0.42–0.94)	<0.001
Serum ferritin	-0.002 (-0.003–-0.0004)	0.009	0.00 (0.00–0.00)	0.405
Alcohol	1.49 (0.10–2.88)	0.036	0.48 (-1.30–2.27)	0.593
Smoking	-2.00 (-4.63–0.62)	0.133	-2.20 (-3.64–-0.77)	0.003
Peritonitis	-2.84 (-4.64–-1.03)	0.002	-1.40 (-2.85–0.05)	0.058
Follow-up month	0.11 (0.02–0.20)	0.014	0.00 (-0.08–0.08)	0.986

Alb, albumin; BMI, body mass index; Coef., regression coefficients; CI, Confidence interval; CRP, C-reactive protein; HIV, human immunodeficiency virus; HB, blood hemoglobin.

^a^Clustered multivariable linear regression model included HIV status, age, race, sex, diabetes, smoking, alcohol use, month of follow-up albumin specimen taken, body mass index, Tenckhoff catheter insertion parameters (site and method, whether laparoscopic or percutaneous), type of primary residence (urban vs rural), highest education level, incidence of peritonitis during follow-up, blood hemoglobin level, and serum levels of bicarbonate, urea, creatinine, ferritin, and C-reactive protein during follow-up

### Mortality

**Fig 2:**
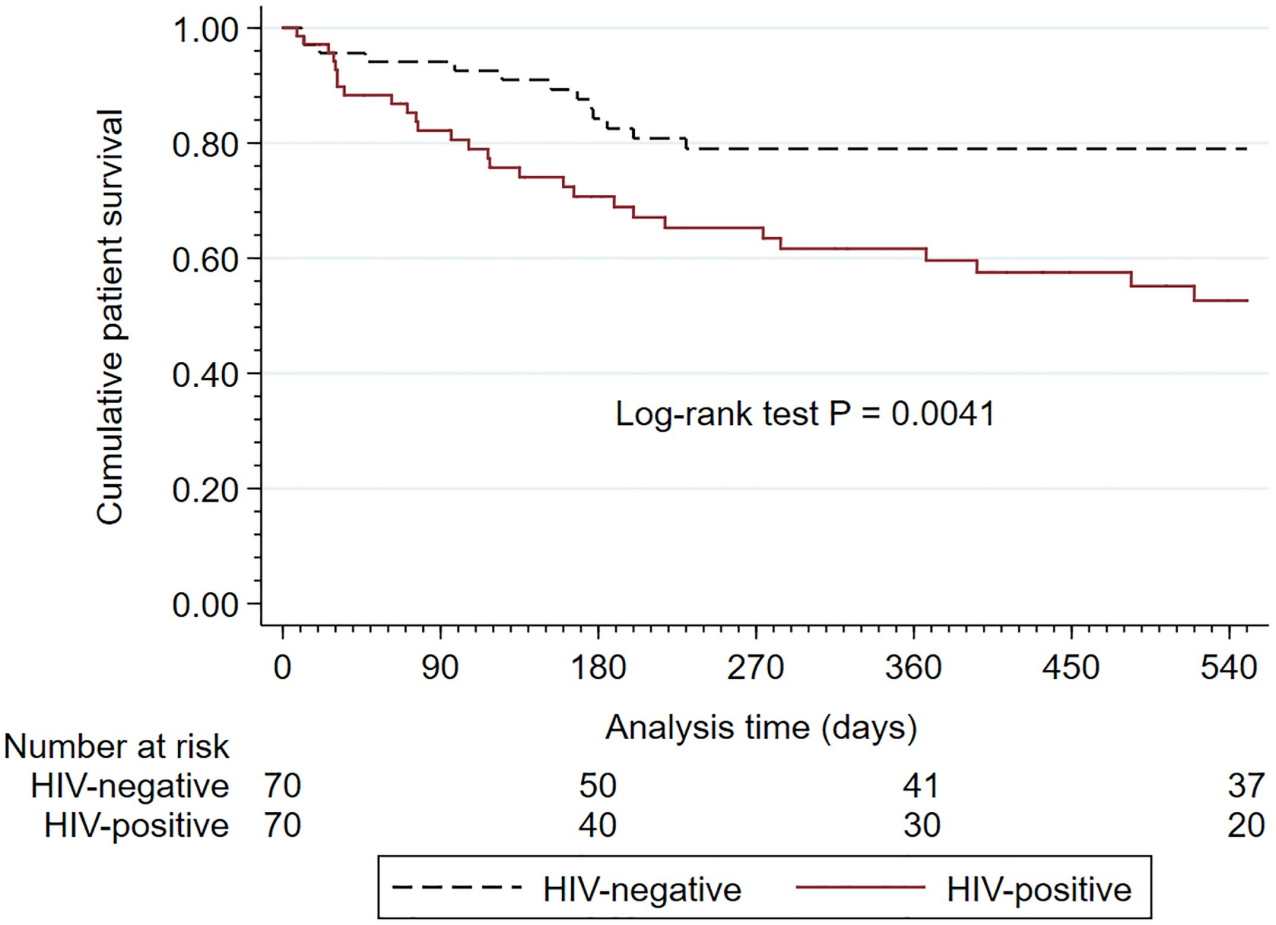
Kaplan–Meier estimates for patient survival censored for catheter loss and loss to follow-up. HIV, human immunodeficiency virus.

After 18 months, all-cause mortality was 18.8% (13/70) in the HIV-negative cohort and 40.0% (28/70) in the HIV-positive cohort (*p* = 0.005), whereas technique failure occurred in 24.3% (17/70) of the HIV-negative cohort and in 27.1% (19/70) of the HIV-positive cohort (*p* = 0.699). Two HIV-negative participants had their Tenckhoff catheters removed due to improved renal function and kidney transplantation, respectively, and were recorded as lost to follow-up. In the HIV-positive cohort, two participants had their Tenckhoff catheters removed due to improved renal function and one participant left voluntarily to undergo private hemodialysis. The profile of the individual causes of death did not differ significantly between the two cohorts (S3 Table). Kaplan–Meier patient survival rates at 18 months censored for technique failure and loss to follow-up were 78.3% (HIV-negative cohort) and 52.5% (HIV-positive cohort), respectively (*p* = 0.004) (Fig 2).

Mortality among patients with baseline serum albumin <25 g/L was 90% (9/10) in the HIV-positive cohort (2.28 deaths/person-years) and 66.7% (2/3) in the HIV-negative cohort (1.03 deaths/person-years). Moreover, 78% of deaths (22/28) in the HIV-positive cohort occurred among patients with baseline serum albumin <35 g/L and 61.5% (8/13) in the HIV-negative cohort. The overall all-cause mortality rate was 0.50 deaths/person-years in the HIV-positive cohort and 0.18 deaths/person-years in the HIV-negative cohort.

On multivariable competitive risk analysis, independent predictors of mortality were baseline CD4+ cell counts <200 cells/μL (adjusted subdistribution hazard ratio [SHR], 3.2; CI, 1.38–7.45; *p* = 0.007), and serum albumin <25 g/L (adjusted SHR, 13.06; CI, 3.09–55.14; *p*<0.001). In the HIV-positive cohort, baseline serum albumin <25 g/L (adjusted SHR, 8.03; CI, 1.70–37.95; *p* = 0.009) and CD4+ cell counts <200 cells/μL (adjusted SHR, 2.79; CI, 1.19–6.58; *p* = 0.019) independently predicted all-cause mortality, whereas in the HIV-negative cohort, baseline serum albumin <30 g/L (adjusted SHR, 64.00; CI, 1.31–3132.56; *p* = 0.036) independently predicted mortality (Table 5).

**Table 5:** Univariate and multivariable competitive risk analysis for cumulative incidence of mortality with technique failure as a competing risk factor.

	Univariate analysis		Multivariable analysis	
	SHR (95% CI)	*p* value	SHR (95% CI)	*p* value
**Combined cohorts**^a^				
HIV	2.48 (1.28–4.79)	0.007	2.19 (0.76–6.30)	0.148
Baseline CD4+ count (cells/μL)				
HIV-negative	Reference		Reference	
CD4+ <200	5.24 (2.22–12.38)	<0.001	3.2 (1.38–7.45)	0.007
CD4+ ≥200	1.99 (0.99–4.02)	0.055	1	
Baseline serum albumin (g/L)				
Alb ≥35	Reference		Reference	
Alb 30–34.9	1.21 (0.46–3.18)	0.705	1.18 (0.28–5.00)	0.820
Alb 25–29.9	2.18 (0.96–4.92)	0.062	1.81 (0.39–8.38)	0.448
Alb <25	8.08 (3.88–16.79)	<0.001	13.06 (3.09–55.14)	<0.001
BMI	1.00 (0.93–1.08)	0.979	0.93 (0.85–1.03)	0.173
Baseline serum CRP	1.01 (1.00–1.01)	0.006	1.00 (0.99–1.01)	0.741
Baseline HB	0.87 (0.74–1.04)	0.120	1.06 (0.84–1.34)	0.606
Baseline serum ferritin	1.001 (1.0003–1.001)	<0.001	1.001 (1.00001–1.001)	0.050
**HIV-positive cohort**^b^				
Baseline serum albumin (g/L)				
Alb ≥35	Reference		Reference	
Alb 30–34.9	0.93 (0.28–3.08)	0.905	0.76 (0.15–3.75)	0.734
Alb 25–29.9	1.06 (0.37–3.00)	0.919	0.76 (0.16–3.61)	0.735
Alb <25	5.35 (2.35–12.20)	<0.001	8.03 (1.70–37.95)	0.009
Baseline CD4+ cell count (cells/μL)				
CD4+ ≥200	Reference		Reference	
CD4+ <200	2.75 (1.23–6.13)	0.013	2.79 (1.19–6.58)	0.019
BMI	1.02 (0.95–1.10)	0.572	0.96 (0.86–1.07)	0.458
Baseline serum CRP	1.01 (1.00–1.01)	0.005	1.00 (0.99–1.01)	0.692
Baseline HB	1.06 (0.87–1.29)	0.561	1.03 (0.77–1.37)	0.866
Baseline serum ferritin	1.001 (1.0005–1.001)	<0.001	1.001 (1.0001–1.001)	0.023
**HIV-negative cohort**^c^				
Baseline serum albumin (g/L)				
Alb ≥35	Reference		Reference	
Alb 30–34.9	1.07 (0.20–5.74)	0.940	3.21 (0.01–1408.64)	0.707
Alb <30	4.74 (1.51–14.93)	0.008	64.00 (1.31–3132.56)	0.036
BMI	0.98 (0.82–1.18)	0.841	0.82 (0.70–0.95)	0.009
Baseline serum CRP	1.00 (0.99–1.01)	0.939	0.98 (0.96–0.99)	0.011
Baseline HB	0.72 (0.54–0.97)	0.029	0.96 (0.42–2.19)	0.928
Baseline serum ferritin	1.00 (0.999–1.001)	0.936	1.00 (1.00–1.00)	0.999

Alb, albumin; BMI, body mass index; CD, cluster of differentiation; CRP, C-reactive protein; HB, hemoglobin; HIV, human immunodeficiency virus; SHR, subdistribution hazard ratio

^a^Multivariable Fine and Gray proportional hazards model for the combined HIV-positive and HIV-negative cohorts included HIV status, baseline serum albumin levels, age, race, sex, smoking status, diabetes diagnosis status, body mass index, Tenckhoff catheter insertion parameters (site and method, whether laparoscopic or percutaneous), type of primary residence (urban vs rural), highest education level, baseline CD4+ cell count, baseline blood hemoglobin level, and baseline serum levels of ferritin and CRP.

^b^Multivariable Fine and Gray proportional hazards model for HIV-positive cohort included baseline serum albumin levels, age, race, sex, smoking, diabetes diagnosis status, body mass index, Tenckhoff catheter insertion parameters (site and method, whether laparoscopic or percutaneous), type of primary residence (urban vs rural), highest education level, baseline CD4+ cell count, baseline blood hemoglobin level, and baseline serum levels of ferritin and CRP.

^c^Multivariable Fine and Gray proportional hazards model for HIV-negative cohort included baseline serum albumin levels, age, race, sex, smoking status, diabetes diagnosis status, body mass index, type of primary residence (urban vs rural), highest education level, baseline blood hemoglobin level, and baseline serum levels of ferritin and CRP.

## Discussion

This prospective cohort study evaluated the effect of HIV infection on serial serum albumin levels and associated mortality in ESRD patients managed with CAPD over an 18-month follow-up period. HIV was associated with significantly lower serum albumin levels at baseline and follow-up and negatively predicted monthly serum albumin levels over 18 months. Moreover, initiation of CAPD was associated with momentary drops in serum albumin levels in both HIV-infected and non-infected ESRD patients. Furthermore, HIV was associated with increased mortality rates, with baseline CD4+ cell counts <200 cells/μL and serum albumin <25 g/L independently predicting all-cause mortality. However, technique failure rates did not significantly differ according to HIV status.

To our knowledge, this is the first report on the effects of HIV-seropositive status on serum albumin levels in ESRD patients managed with CAPD. The HIV-positive cohort consistently had lower monthly serum albumin levels compared to the HIV-negative cohort during follow-up. The negative influence of HIV on serum albumin levels in these patients was also reaffirmed on linear regression analysis, where after adjustments, HIV-positive status remained a significant negative predictor of monthly serum albumin levels during follow-up. HIV-positive patients initiated on CAPD already presented with a mean serum albumin level below 35 g/L with only 25.7% of the HIV-positive cohort having serum albumin levels at or above this threshold, while 58.6% of the HIV-negative cohort had levels ≥35 g/L at baseline. This baseline handicap is suspected to be partially attributable to uncontrolled HIV as nearly half of the HIV-positive cohort was started on ART within 6 months before the initiation of CAPD. However, this HIV-associated negative effect on serum albumin appeared to extend beyond HIV control as over half of the patients with suppressed viral loads and those with CD4+ cell counts ≥350 cells/μL also had serum albumin levels below 35 g/L at the start of CAPD. HIV is thought to influence serum albumin levels through a number of mechanisms such as chronic inflammation, infections, nutritional deficiencies, chronic gastrointestinal ailments, altered metabolism and absorption of vital nutrients, nutrient-drug interactions, and increased caloric requirements [31–34].

Furthermore, on linear regression, serum CRP and blood hemoglobin levels were significant predictors of serum albumin levels, which is a result highlighting the prominent role played by inflammation in the suppression of serum albumin levels, particularly in the HIV-positive cohort. Significantly increased levels of inflammatory markers were noted at baseline among those who were HIV positive and among those who had serum albumin levels <35 g/L in the combined cohort, reiterating the importance of inflammation in influencing serum albumin changes. The baseline serum albumin handicap associated with HIV appear to persist throughout most of the follow-up period, maintaining a stable mean difference range that did not improve substantially on continued treatment with ART. A report by Thuppal et al. [27] from the National Health and Nutrition Examination Survey (NHANES) data of a non-CAPD general population sample in the United States also described lower serum albumin levels in HIV-positive participants on ART than in non-infected NHANES participants. However, the serum albumin levels of the NHANES HIV-positive cohort were much higher (41.7 g/L for men and 38.6 g/L for women) than the levels observed for our HIV-positive cohort, which probably reflects the added contributions of underlying renal disease, dialysis treatment, and poor socioeconomic conditions endemic to sub-Sahara Africa. Persistence of hypoalbuminemia and malnutrition in patients on ART has been reported by other studies in sub-Sahara Africa, highlighting the ineffectiveness of ART alone to adequately mitigate these challenges [17, 18].

Both HIV-positive and HIV-negative cohorts experienced decreases in monthly serum albumin levels on initiation of CAPD peaking at 12%–13% below the baseline mean values by the 4th month after the insertion of the Tenckhoff catheter. The rates of decline of serum albumin levels in the two cohorts were not significantly different. This comparable serum albumin reduction in both cohorts was likely due to dialysate losses and inadequate compensatory mechanisms to re-establish baseline albumin homeostasis. Both cohorts showed a similar recovery of monthly mean serum albumin levels until the 18-month follow-up with mean albumin values 1%–2% below baseline mean levels. However, paired t-test comparison values were consistently lower than the baseline values throughout the follow-up period in the HIV-negative cohort. The 38 patients in the HIV-negative cohort who had a patent catheter until 18 months had a mean serum albumin difference of 2.26 ± 6.28 g/L compared to baseline values at the initiation of CAPD. This observation highlights that the survival benefit of those with higher serum albumin reaching 18 months was disproportionately higher compared to those with lower albumin values and shows a sustained drop in serum albumin levels that was inadequately compensated for at the end of the 18-month follow-up period. In the HIV-positive cohort, mean serum albumin levels were significantly lower than the baseline values in the first 13 months of follow-up. After 13 months, the HIV-positive cohort had mean serum albumin levels that were very close to baseline levels, but paired t-test comparison yielded differences that were not statistically significant. The failure to reach statistical significance in these paired differences may have been influenced by the higher incidence of deaths in the HIV-positive cohort, which disproportionately affected patients with low serum albumin levels and conspicuously compromised the remaining sample size, thereby obscuring any significant differences that could have been observed otherwise. Notably, albumin losses in dialysis are compensated by decreases in albumin catabolism and increases in hepatic synthesis to maintain serum albumin homeostasis [14, 35]. However, the effectiveness of compensatory mechanisms can be compromised by chronic undernutrition, chronic inflammation, and overhydration [36, 37]. Although the design of this study did not allow discriminating between or properly account for all these factors, the close relationship between the observed inflammatory markers and serum albumin levels in this study suggests that inflammation had a substantial role in influencing the observed outcomes.

The HIV-positive cohort was associated with an increased rate of death, but both cohorts experienced very similar technique failure rates. On multivariable competitive risk analysis, consistent independent predictors of death were baseline serum albumin levels <25 g/L and CD4+ cell counts <200 cells/μL. These predictors highlight the importance of immunocompetence and nutritional-inflammatory factors in influencing mortality risk in CAPD. The predictive potential of serum albumin and the interplay between malnutrition and inflammation is well recognized and has been shown to strongly predict mortality [23, 38–40]. Chronic inflammation has been suggested to induce hypoalbuminemia through inhibition of albumin synthesis and stimulation of its catabolism [41, 42]. The predictive value of baseline serum albumin was strongly underscored in our HIV-positive cohort where 90% of patients with baseline serum albumin levels <25 g/L died during follow-up, and baseline values below this threshold were strongly predictive of mortality (SHR 8.03, *p* = 0.009). In the HIV-negative cohort, serum albumin levels <30 g/L independently predicted mortality events (SHR 64, *p* = 0.036). Similarly, in their retrospective analysis of 12,171 general PD patients, Mehrotra et al. found that baseline serum albumin levels below 30 g/L were associated with a threefold increase in the adjusted mortality risk [40]. Thus, we postulate that the baseline serum albumin level is a stable marker of malnutrition-inflammatory syndrome and holds robust predictive utility for risk of mortality in both HIV-infected ESRD patients initiated on CAPD as in HIV non-infected patients. This marker can be used to prognosticate and guide management with special emphasis on measures that improve protein energy nutrition and inflammation in those at increased risk.

This study has limitations. First, this study has single-center observational study design which inherently limits the causality that can be inferred from observed associations. Second, there was unavailability of peritoneal equilibration test (PET) results, and the lack of peritoneal membrane transporter characteristics for a significant portion of patients in our cohort prohibited the incorporation of this variable in our models. There was increased mortality in the HIV-positive cohort particularly in the first 6 months of follow-up that has been described elsewhere [29], which limited the number of study participants reaching this milestone at which PETs were conducted. Although transporter status has been reported to influence serum albumin levels and mortality [43], its omission is not expected to have significantly affected the observed associations. Furthermore, the high dropout rate in the HIV-positive cohort may have limited our ability to appreciate significant differences in mean serum albumin levels beyond 13 months. Lastly, the use of an amino acid-based peritoneal dialysis solution was not recorded during follow-up. However, only two patients at baseline and six more during follow-up had recorded serum albumin levels below the local threshold of 20 g/L determining eligibility to start amino acid-based CAPD fluids in our hospital, a policy based on economic considerations. Hence, the small number of patients likely to have had access to these CAPD fluids precludes any probable material effect this variable would have had on the observed results.

### Conclusions

This study indicates that HIV infection can adversely influence serum albumin levels, which in turn may contribute to the increased mortality risk observed in ESRD patients managed with CAPD. It is proposed that the mortality risk attributable to HIV infection may be substantially influenced by a malnutrition-inflammatory syndrome, and low baseline serum albumin levels appear to reliably predict its manifestation. Measures directed at improving nutrition and mitigating factors contributing to chronic inflammation can help minimize this risk.

## Supporting information

S1 Fig Linear regression curves of serum albumin levels according to HIV. β_0–4_, linear regression slope for serum albumin levels between baseline and 4th month β_4–18_, linear regression slope for serum albumin levels between the 4th and 18th month Δβ_0–4 (HIV-negative—HIV-Positive)_, difference in linear regression slopes of serum albumin levels between the HIV-negative and HIV-positive cohorts, for the period between baseline and the 4th month Δβ_4–18 (HIV-negative—HIV-Positive)_, difference in linear regression slopes of serum albumin levels between the HIV-negative and HIV-positive cohorts, for the period between the 4th and 18th month
(TIF)

S1 Table Comparison of mean serum albumin levels according to HIV status. CI, Confidence interval; HIV, human immunodeficiency virus; MD, mean difference; SD, Standard deviation.
(PDF)

S2 Table Paired mean serum albumin differences from the baseline. CI, Confidence interval; HIV, human immunodeficiency virus; MD, mean difference; SD, Standard deviation.
(PDF)

S3 Table Listed causes of mortality events. CVS, Cardiovascular disease; HIV, human immunodeficiency virus. ^a^Fisher’s exact test.
(PDF)
