# Supplementary figures and images for: Serum albumin and mortality in patients with HIV and end-stage renal failure on peritoneal dialysis
Source: PLoS One. 2019 Jun 10;14(6):e0218156. doi: 10.1371/journal.pone.0218156 (PMC6557525; doi:10.1371/journal.pone.0218156)

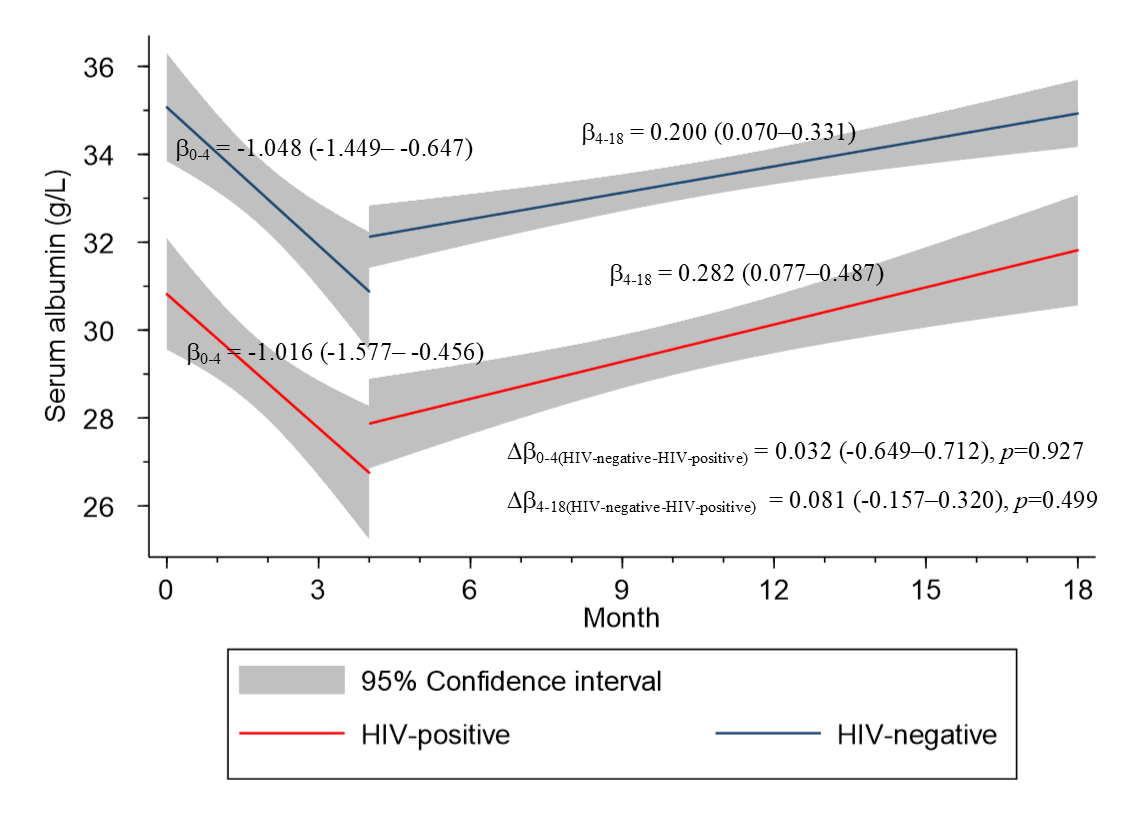

Supplement: S1 Fig — β0–4, linear regression slope for serum albumin levels between baseline and 4th month β4–18, linear regression slope for serum albumin levels between the 4th and 18th month Δβ0–4 (HIV-negative—HIV-Positive), difference in linear regression slopes of serum albumin levels between the HIV-negative and HIV-positive cohorts, for the period between baseline and the 4th month Δβ4–18 (HIV-negative—HIV-Positive), difference in linear regression slopes of serum albumin levels between the HIV-negative and HIV-positive cohorts, for the period between the 4th and 18th month (TIF) [file pone.0218156.s001.tif]
