# Supplementary material for: Serum albumin and mortality in patients with HIV and end-stage renal failure on peritoneal dialysis
Source: PLoS One. 2019 Jun 10;14(6):e0218156. doi: 10.1371/journal.pone.0218156 (PMC6557525; doi:10.1371/journal.pone.0218156)
Supplement: S1 Table — CI, Confidence interval; HIV, human immunodeficiency virus; MD, mean difference; SD, Standard deviation. (PDF) [file pone.0218156.s002.pdf]

S1 Table. Comparison of mean serum albumin levels according to HIV status

Visit month	Negative HIV		Positive HIV		Independent t test	
	N	Mean serum albumin (g/L) ± SD	N	Mean serum albumin (g/L) ± SD	MD (95% CI)	*p* value
Baseline	70	35.3 ± 6.73	70	31.0 ± 6.56	4.24 (2.02–6.46)	0.0002
1st month	59	33.9 ± 6.49	59	29.9 ± 6.19	4.03 (1.72–6.35)	0.0008
2nd month	52	32.4 ± 6.19	47	27.7 ± 7.14	4.68 (2.02–7.34)	0.0007
3rd month	53	32.1 ± 6.14	42	28.2 ± 7.17	3.96 (1.25–6.68)	0.0046
4th month	53	31.0 ± 6.85	43	27.0 ± 6.22	4.01 (1.33–6.69)	0.0037
5th month	42	31.7 ± 5.89	39	27.7 ± 6.80	4.04 (1.24–6.85)	0.0053
6th month	45	32.3 ± 4.73	39	28.9 ± 7.30	3.39 (0.76–6.03)	0.0122
7th month	43	33.7 ± 4.73	30	30.0 ± 6.90	3.71 (0.99–6.42)	0.0081
8th month	41	34.0 ± 4.27	35	29.4 ± 5.80	4.49 (2.19–6.80)	0.0002
9th month	36	34.0 ± 4.25	34	30.1 ± 5.46	3.88 (1.55–6.21)	0.0014
10th month	38	34.0 ± 4.23	32	28.6 ± 5.89	5.37 (2.95–7.79)	<0.0001
11th month	40	33.8 ± 4.61	28	29.2 ± 5.30	4.65 (2.23–7.06)	0.0003
12th month	40	33.7 ± 5.57	30	29.9 ± 5.95	3.77 (1.00–6.53)	0.0083
13th month	36	33.6 ± 4.85	27	30.7 ± 6.34	2.88 (0.06–5.69)	0.0452
14th month	38	33.7 ± 4.39	23	31.1 ± 5.84	2.55 (-0.08–5.19)	0.0572
15th month	37	33.9 ± 4.56	21	31.4 ± 6.16	2.51 (-0.33–5.35)	0.0819
16th month	36	34.5 ± 4.17	21	31.1 ± 7.17	3.36 (0.35–6.36)	0.0291
17th month	36	34.6 ± 4.32	21	31.8 ± 6.95	2.77 (-0.22–5.84)	0.0680
18th month	38	34.7 ± 3.72	20	30.7 ± 6.96	3.99 (1.19–6.79)	0.0061

CI, Confidence interval; MD, mean difference; SD, Standard deviation; HIV, human immunodeficiency virus.
