# Supplementary material for: Serum albumin and mortality in patients with HIV and end-stage renal failure on peritoneal dialysis
Source: PLoS One. 2019 Jun 10;14(6):e0218156. doi: 10.1371/journal.pone.0218156 (PMC6557525; doi:10.1371/journal.pone.0218156)
Supplement: S2 Table — CI, Confidence interval; HIV, human immunodeficiency virus; MD, mean difference; SD, Standard deviation. (PDF) [file pone.0218156.s003.pdf]

S2 Table. Paired mean serum albumin differences from the baseline

**Paired t test**	**HIV-negative**				**HIV-positive**			
	**N**	**MD ± SD**	**95% CI**	***p* value**	**N**	**MD ± SD**	**95% CI**	***p* value**
Baseline	Reference				Reference			
1st month	59	1.46 ± 5.20	0.10–2.81	0.0354	59	1.88 ± 5.13	0.54– 3.22	0.0066
2nd month	52	3.54 ± 5.96	1.88–5.20	0.0001	47	4.47 ± 5.97	2.72–6.22	<0.0001
3rd month	53	4.13 ± 5.73	2.55–5.71	<0.0001	42	4.38 ± 6.98	2.20–6.56	0.0002
4th month	53	3.89 ± 5.83	2.28–5.49	<0.0001	43	5.35 ± 7.81	2.95–7.75	0.0001
5th month	42	4.24 ± 5.50	2.52–5.95	<0.0001	39	5.15 ± 7.80	2.62–7.68	0.0002
6th month	45	4.27 ± 5.87	2.50–6.03	<0.0001	39	3.79 ± 7.85	1.25–6.34	0.0045
7th month	43	2.95 ± 6.41	0.98–4.93	0.0043	30	2.43 ± 7.44	-0.34–5.21	0.0835
8th month	41	2.63 ± 6.10	0.71–4.56	0.0086	35	3.34 ± 6.88	0.98–5.70	0.0069
9th month	36	2.14 ± 7.18	-0.29–4.57	0.0825	34	2.82 ± 6.51	0.55–5.10	0.0164
10th month	38	2.74 ± 6.40	0.63–4.84	0.0121	32	4.59 ± 6.05	2.41–6.77	0.0002
11th month	40	2.60 ± 6.11	0.65–4.55	0.0104	28	4.50 ± 6.80	1.86–7.14	0.0016
12th month	40	3.05 ± 6.88	0.85–5.25	0.0078	30	3.37 ± 7.34	0.63–6.11	0.0178
13th month	36	3.56 ± 6.88	1.23–5.88	0.0038	27	3.04 ± 7.11	0.23–5.85	0.0353
14th month	38	3.26 ± 6.71	1.06–5.47	0.0049	23	2.48 ± 7.34	-0.69–5.65	0.1194
15th month	37	3.16 ± 6.84	0.88–5.44	0.0079	21	1.76 ± 6.57	-1.23–4.75	0.2335
16th month	36	3.06 ± 7.29	0.59–5.52	0.0157	21	1.81 ± 6.82	-1.29–4.91	0.2379
17th month	36	2.58 ± 6.66	0.33–4.84	0.0260	21	1.38 ± 6.87	-1.74–4.51	0.3677
18th month	38	2.26 ± 6.28	0.20–4.33	0.0325	20	2.60 ± 7.15	-0.74–5.94	0.1203

CI, Confidence interval; MD, mean difference; SD, Standard deviation; HIV, human immunodeficiency virus.
