# Supplementary material for: Serum albumin and mortality in patients with HIV and end-stage renal failure on peritoneal dialysis
Source: PLoS One. 2019 Jun 10;14(6):e0218156. doi: 10.1371/journal.pone.0218156 (PMC6557525; doi:10.1371/journal.pone.0218156)
Supplement: S3 Table — CVS, Cardiovascular disease; HIV, human immunodeficiency virus. aFisher’s exact test. (PDF) [file pone.0218156.s004.pdf]

S3 Table. Listed causes of mortality events

**Cause of death**	**HIV-negative (n = 70)**	**HIV-positive (n = 70)**	***p*-value**
Peritonitis/tunnel infection, n (%)	3 (4.3%)	4 (5.7%)	0.693[a]
Sepsis without peritonitis, n (%)	1 (1.4%)	7 (10%)	
CVS death, n (%)	1 (1.4%)	3 (4.3%)	
Home death, n (%)	8 (11.4%)	13 (18.6%)	
Gastroenteritis, n (%)	0	1 (1.4%)	

CVS, cardiovascular disease; HIV, human immunodeficiency virus.

[a]Fisher's exact test
